# Monoclonal gammopathy of renal significance in western China: A large cohort study dominated by amyloidosis with distinct clinical outcomes

**DOI:** 10.1016/j.clinsp.2026.100866

**Published:** 2026-02-10

**Authors:** Junru Wang, Kun Peng, Guisen Li, Shasha Chen

**Affiliations:** Department of Nephrology and Institute of Nephrology, Sichuan Academy of Medical Sciences & Sichuan Provincial People’s Hospital, University of Electronic Science and Technology of China, Sichuan Clinical Research Center for Kidney Diseases, Chengdu, China

**Keywords:** Monoclonal gammopathy of renal significance, MGRS, Renal amyloidosis, AL amyloidosis, Prognostic factors, Kidney biopsy, Cohort study, China

## Abstract

**Aim:**

To characterize the epidemiology, clinicopathological features, and renal outcomes in patients with Monoclonal Gammopathy of Renal Significance (MGRS).

**Methods:**

The authors conducted a retrospective analysis of all renal biopsy-confirmed MGRS cases diagnosed between 2010‒2020, with histopathological review by two independent pathologists. Post-hoc power analysis confirmed adequate statistical power (92 %).

**Results:**

Among 124 histologically confirmed MGRS cases (median age 64.0-years, IQR 52.5‒68.0), renal amyloidosis predominated (75.8 %), followed by monoclonal immunoglobulin deposition disease (MIDD, 10.5 %), cryoglobulinemic GN (4.8 %), proliferative GN with monoclonal Ig deposits (PGNMID, 2.4 %), light chain proximal tubulopathy (3.2 %), and C3 glomerulopathy with monoclonal gammopathy (1.6 %). Amyloidosis patients primarily presented with nephrotic syndrome (77.7 %), while fewer exhibited acute kidney injury (3.2 %), chronic kidney disease (16.1 %), or ESRD (6.7 %). Compared to other MGRS subtypes, amyloidosis patients demonstrated significantly lower anemia rates (*p* < 0.001), higher LDH (*p* = 0.035), preserved eGFR (*p* < 0.001), greater proteinuria (*p* = 0.037), and hypoalbuminemia (*p* < 0.001). The renal response rates were 26.1 % (Amyloidosis-Associated MGRS, MGRS-A) versus 33.3 % (Non-Amyloidosis MGRS, MGRS-NA), while hematologic responses were 8.7 % versus 0 %, respectively. Both hematologic (*p* = 0.007) and renal responses (*p* = 0.009) correlated with improved survival. MGRS-A showed inferior renal survival (*p* = 0.05). Multivariate analysis identified hypotension (*p* = 0.005), elevated creatinine (*p* = 0.002), and cardiac involvement (*p* = 0.022) as independent predictors of ESKD, while age (*p* < 0.001) and cardiac involvement (*p* < 0.001) predicted mortality.

**Conclusion:**

MGRS represents a clinically significant cause of kidney injury in monoclonal gammopathy patients, with amyloidosis being the predominant etiology. MGRS-A portends a worse prognosis than MGRS-NA. Therapeutic responses in both hematologic and renal parameters predict survival benefits. Age and cardiac involvement emerge as key prognostic markers for both renal and patient survival.

## Highlights

• Renal amyloidosis is the dominant lesion in this Chinese MGRS cohort.
• Amyloidosis MGRS shows a distinct clinical phenotype at presentation.
• Cardiac involvement is a key predictor of end-stage kidney disease.
• Therapy response (hematologic/renal) is linked to better survival.
• Early clone-directed therapy is critical for amyloidosis MGRS.

## Introduction

Monoclonal Gammopathy of Renal Significance (MGRS) is a clonal disorder that represents a group of renal lesions caused by a paraprotein secreted by a clone of mature B lymphocytes or plasma cells and not meeting diagnostic criteria for an overt hematological malignancy, with increasing risk of progression to End Stage Renal Disease (ESRD) and the underlying hematologic malignancy.^1^, ^2^, ^3^, ^4^ MGRS is commonly underdiagnosed due to its rarity and lack of familiarity. The spectrum of MGRS associated renal lesions contained a various pathological patterns, it can be categorized as organized, non-organized and non-immunoglobulin according to the ultrastructural features by electron microscopy and immunofluorescence findings on renal deposits,^5^^,^^6^ i.e., immunotactoid glomerulonephritis, C3 glomerulopathy, Proliferative Glomerulonephritis with Monoclonal Immunoglobulin Deposits (PGNMID); the tubular compartment, i.e., Light-Chain Proximal Tubulopathy (LCPT); and the multiple compartments, i.e., immunoglobulin-related amyloidosis, monoclonal immunoglobulin deposition disease, and Thrombotic Microangiopathy (TMA)-pattern as indirect paraprotein-mediated endothelial injury according to a consensus report. Immunoglobulin light chain (AL) amyloidosis is the most common type of MGRS, which is a heterogeneous and life-threatening disease characterized by the deposition of amyloid fibrils derived from misfolded light chains.^7^ The literature lacks the incidence, clinical features, and prognosis of MGRS from Western China. The objective of this study was to report a large retrospective MGRS case series study from the single institute and analyze the spectrum of MGRS and prognosis. Owing to the high rate of AL amyloidosis within MGRS, the authors grouped this cohort into amyloidosis and non-amyloidosis MGRS.

## Materials and methods

Patients with MGRS who were newly diagnosed by renal biopsy in the studied center from January 2010 to December 2020 were included in the present study. Inclusion criteria for this study were: 1) Age ≥ 18-years; 2) Positive serum immunofixation electrophoresis for monoclonal immunoglobulin. The exclusion criteria were as follows: 1) Patients with Monoclonal Gammopathy of Undetermined Significance (MGUS) and renal diseases not related to M protein; 2) Patients who suffered from membranous nephropathy, IgA nephropathy, or other confounding diseases based on relevant clinical and pathological information were excluded.

All patients included in the analysis required local hemato-pathological confirmation by renal biopsy for one of the following pathological diagnoses: AL amyloidosis, Monoclonal Immunoglobulin Deposition Disease (MIDD), proliferative Glomerulonephritis (GN) with Monoclonal Immunoglobulin Deposition Disease (PGNMID), monoclonal fibrillary GN, immunotactoid GN, cryoglobulinemic GN, Light Chain Proximal Tubulopathy (LCPT), crystal-storing histiocytosis, C3 glomerulopathy with monoclonal gammopathy, and thrombotic microangiopathy.^8^, ^9^, ^10^, ^11^, ^12^, ^13^, ^14^ The diagnosis of systemic AL amyloidosis was confirmed by the presence of apple-green birefringence under polarized light after Congo red staining and the identification of fibrils with a diameter of 8–12 nm by electron microscopy.^15^ Clinicopathologic and follow-up data were obtained from the electronic medical record system. The protocol for this study was approved by the medical ethics committee of the Sichuan Academy of Medical Sciences and Sichuan Provincial People’s Hospital (Approval n° 2025,336), and informed written consent for the treatment they received was obtained from all of the identified patients. All treatment studies were performed in accordance with relevant guidelines and regulations. This retrospective cohort study was reported in accordance with the Strengthening the Reporting of Observational Studies in Epidemiology (STROBE) Statement.

### Clinical and laboratory data

Baseline clinical data included gender, age, disease duration, blood pressure (measured on the morning of the biopsy day), and gross hematuria at the time of biopsy. Laboratory data included 24 h urinary protein excretion (Upr), serum Albumin (Alb), Serum creatinine (Scr), Uric Acid (UA), liver enzymes, and estimated Glomerular Filtration Rate (eGFR) calculated using the equation of CKD-EPI (CKD Epidemiology Collaboration) formula. Scr and Upr during follow-up, heavy and light chain isotype, serum-free light-chain κ and λ, the percentage of clonal plasma cells in bone marrow, and the presence of B-cell lymphoproliferative disease is also collected.

### Renal pathology

All patients underwent ultrasound-guided percutaneous renal biopsy. Biopsy specimens were processed for Light Microscopy (LM), IF and Electron Microscopy (EM). Frozen tissues were subjected to IF staining, including IgG, IgA, IgM, C3, C1q, κ, and λ light chain staining, and formalin-fixed tissues were subjected to IF staining, including fibronectin staining. For LM, samples from all patients were stained with hematoxylin and eosin, Periodic Acid-Schiff (PAS), Masson trichrome, and PASM-Masson.

### Treatment

Treatments were divided into proteasome inhibitor-based (bortezomib, carfilzomib or ixazomib, i.e., PI), Immunomodulatory (IMiD) drug-based (thalidomide, lenalidomide, pomalidomide, i.e., IMiD), monoclonal antibody-based (daratumumab, rituximab, i.e., MoA), corticosteroids (prednisone, dexamethasone), chemotherapy-based (chemotherapy alone), and Autologous Stem Cell Transplantation (ASCT).

### Follow-up and outcome evaluation

Hematologic response was defined as a Complete Response (CR) if normalization of FLC was obtained when available. Otherwise, a disappearance of monoclonal protein at electrophoresis and with serum or urinary immunofixation or disappearance of the plasma cellular clone. Defining Very Good Partial Response (VGPR) and Partial Response (PR) required 90 % or 50 % monoclonal protein reduction, respectively.^16^^,-^^17^

Renal response was assessed as a reduction of > 30 % of 24 h proteinuria (in the absence of renal progression defined by progressive decrease of > 25 % of eGFR).^18^

### Statistical analysis

All data were analyzed using the statistical software SPSS 19.0. Normally distributed variables were expressed as mean ± SD. Non-parametric variables were expressed as median and interquartile range. Categorical variables were expressed in percentages. All parameters were compared by Chi-square test, Fisher test for categorical data, and the Student’s *t*-test, one-way analysis of variance (ANOVA) analysis, or Kruskal-Wallis test for continuous data. Survival rate was estimated with the Kaplan-Meier method. The outcome event was defined as a combined renal endpoint: 30 % reduction in eGFR or ESRD. Cox proportional hazards models were used for survival analyses. Results are presented as Hazard Ratios (HR) and Odds Ratios (OR) with 95 % Confidence Intervals, respectively; *p* < 0.05 was considered statistically significant.

## Results

### Demographic and disease characteristics

**Fig. 1:**
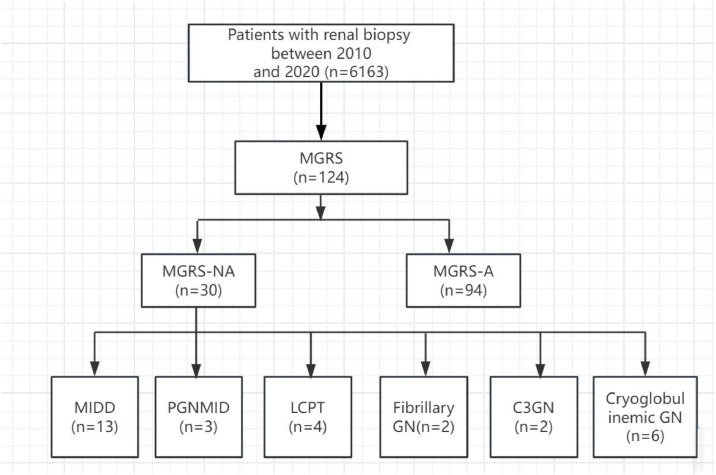
Study flow chart of patients with MGRS who underwent kidney biopsies and pathological spectrum from 2010 to 2020.

Table 1 summarizes the patients' characteristics at presentation. Out of 6163 renal biopsies obtained during the study period, 124 biopsy-proven MGRS cases were encountered during the studied period. There were 71 males (57.3 %) and 53 females (42.7 %) with a median age of 64.0 (52.5‒68.0) ys, accounting for 2.0 % of renal biopsies in the studied institute. The most common MGRS-related lesion was renal amyloidosis (75.8 %), followed by Monoclonal Immunoglobulin Deposition Disease (MIDD) (10.5 %), Cryoglobulinemic GN (4.8 %), Proliferative Glomerulonephritis with Monoclonal Immunoglobulin Deposits (PGNMID) (2.4 %), LCPT (3.2 %), and C3 glomerulopathy with monoclonal gammopathy (1.6 %). IgG kappa was the most frequently identified immunoglobulin in renal biopsies. In amyloidosis patients, the most frequent manifestations at the time of diagnosis were nephrotic syndrome (77.7 %), acute kidney injury (3.2 %), chronic kidney disease (16.1 %), and end-stage renal disease (6.7 %). At presentation, edema, hypotension, dyspnea and fatigue were common clinical signs. It was notable that 25 % patients had cardiac, 6.7 % had neurological disease involvement in amyloidosis patients. MGRS-A group had a higher rate of patients presenting with nephrotic syndrome, a lower rate of acute kidney injury and a lower rate of chronic kidney disease (*p* < 0.001) (Fig. 1; Table 1).

**Table 1:** Patient's clinical characteristics in MGRS.

	MGRS-A (*n* = 94)	MGRS-NA (*n* = 30)	p-value
**Median age (range), years**	65 (54‒68)	57 (50‒67)	0.167
**Male sex, n (%)**	58 (61.7 %)	13 (43.3 %)	0.077
**Diabetes mellitus, n (%)**	3 (5 %)	0 (0)	0.099
**CKD, n (%)**			0.442
Stage 3	8 (13.6 %)	3 (33.3 %)	
Stage 4	4 (6.8 %)	3 (33.3 %)	
Stage 5	1 (1.7 %)	2 (22.2 %)	
**Organ’s involvement, n (%)**			
Heart	25.0	11.1	0.357
Liver	6.7	11.1	0.506
Neuro	8.3	0	0.062
Digestive tract	8.3	0	0.202
Clinical signs, n (%)			0.355
Edema	47.9	83.3	
Arterial hypotension	38.3	0	
Hypertension	22.3	70	
Dyspnea	2.1	0	
Fatigue	10.6	16.7	
Disorder of consciousness	1.1	0	
**Biology**			<0.001
Nephrotic syndrome	77.7	13.3	
AKI	3.2	20.0	
CKD	16.1	63.5	
ESRD	6.7	3.2	
**Type of MGRS**			<0.001
MIDD	0	13	
PGNMID	0	3	
LCPT	0	4	
Fibrillary Glomerulonephritis	0	2	
C3GN	0	2	
Cryoglobulinemic GN		6	
Amyloidosis	94	0	

eGFR, estimated Glomerular Filtration Rate; MGRS-A, Amyloidoid-Associated Monoclonal Gammaglobulinemia of Renal Significance; MGRS-NA, Non-Amyloidosis-Associated Gammaglobulinemia of Renal Significance; PGNMID, Proliferative Glomerulonephritis with Monoclonal Immunoglobulin Deposits; MIDD, Monoclonal Immunoglobulin Deposition Disease, LCPT, Light Chain Proximal Tubulopathy, Cryoglobulinaemic GN, Cryoglobulinaemic Glomerulonephritis; C3GN, C3 Glomerulonephritis,.

### Laboratory characteristics

Laboratory results at presentation are shown in Table 2. Patients with amyloidosis had a lower rate of anemia (*p* < 0.001), higher levels of LDH (*p* = 0.035), eGFR (*p* < 0.001), and 24-hour urinary protein (*p* = 0.037), and a lower serum albumin (*p* < 0.001). No significant differences were found in free light chain ratio between the two groups. For the patients who underwent bone marrow biopsy, the authors found that patients with amyloidosis had a lower plasma cell percentage (0.016), were less likely to have a plasma cell percentage over 10 % (*p* = 0.005), and were less likely to have an abnormal bone marrow biopsy specimen (Table 2).

**Table 2:** Patient's laboratory characteristics in MGRS.

	MGRS-A (*n* = 94)	MGRS-NA (*n* = 30)	p-value
**Serum studies**			
Hemoglobin, g/dL	130.5 ± 21.2	94.9 ± 18.8	<0.001
LDH U/L	279.5 ± 10.2	164.1 ± 21.5	0.035
Albumin (mg/dL), n (%)	24.2 ± 7.2	37.3 ± 6.8	<0.001
Creatinine (mg/dL)	100.9 ± 6.5	408.5 ± 42.4	0.074
eGFR (mL/min/1.73 m^2^)	75.9 ± 29.7	31.6 ± 29.2	<0.001
β−2-microglobulin (mg/L)	3.5 ± 1.5	16.2 ± 8.5	0.18
TnI	20.5 (3.6‒82.4)	4.9 (0‒16.3)	0.184
BNP	233.7 ± 24.5	358.5 ± 49.1	
**Urinary studies**			
24 h urine protein (g)	6.0 (3.5‒8.8)	1.9 (1.5‒5.5)	0.037
Hematuria, n (%)			
**Hematologic studies**			
FLC κ, (mg/dL), median* (range)	24.1 (16.9‒31.6)	152.0 (38.3‒340.0)	0.234
FLC λ, median* (range)	103.0 (46.1‒183.5)	42.9 (38.5‒114.0)	0.429
**Immunofixation electrophoresis, n (%)**			0.01
IgG λ %	56.4	56.4	
IgG κ %	2.1	0	
IgA λ %	17	10	
IgA κ %	0	13.3	
IgM λ %	2.1	0	
IgM κ %	1.1	0	
κ %	1.1	23.3	
λ %	17	10	
IgG κ + IgM κ %	1.1	0	
IgD/IgE %	2.1	0	
Bone marrow involvement ( % PCs), median* (range)	3.5 (2.0‒5.5)	8.0 (3.5‒12.5)	0.016
Plasma cell > 10 %, n (%)	10 (10.6 %)	10 (33.3 %)	0.005

FLC, Free Light Chains.

### Treatment and response

**Fig. 2:**
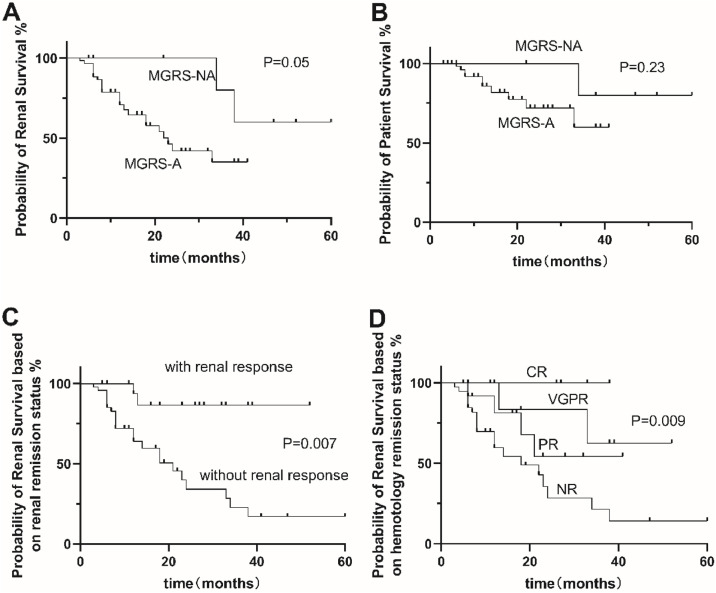
Overall renal (A) and patient (B) survival of Amyloidosis-Associated Monoclonal Gammopathy of Renal Significance (MGRS-A) and Non-Amyloidosis-Associated Gammopathy of Renal Significance (MGRS-NA). Overall renal survival in the groups with amyloidosis-associated monoclonal gammopathy of renal significance and non-amyloidosis-associated gammopathy of renal significance depending on renal response (C) and hematological response (D). Notes, Pairwise log-rank p-values of Fig. 2D are indicated for comparisons between response groups. Significant differences were observed between NR and CR (*p* = 0.016), NR and VGPR (*p* = 0.024), and NR and PR (*p* = 0.016). No other comparisons reached statistical significance (*p* > 0.05). CR, Complete Response; VGPR, Very Good Partial Response; PR, Partial Response; NR, No Response.

Twenty-five patients (26.6 %) in the MGRS-A group were treated with first-line plasma cell clone-directed chemotherapy (including bortezomib, melphan, lenalidomide, and thalidomide) and/or stem cell transplantation, 10 patients with HDM/ASCT (melphalan/autologous hematopoietic stem cell transplantation). The overall renal response rate was 26.1 % and 33.3 % in the MGRS-A and MGRS-NA groups, respectively. The overall hematology response rate was 8.7 % and 0 % in the MGRS-A and MGRS-NA groups, respectively (Table 3). Survival analysis was performed between amyloidosis-associated Monoclonal Gammopathy of Renal Significance (MGRS-A) and Non-Amyloidosis-Associated Gammopathy of Renal Significance (MGRS-NA). MGRS-A had lower renal survival than the MGRS-NA group (*p* = 0.05). Both hematologic and renal responses were associated with longer survival. In the MGRS group, patients with renal response and hematological response had a higher rate of renal survival than those without renal response and hematological response (*p* = 0.007, *p* = 0.009) (Fig. 2).

**Table 3:** Treatment and response in MGRS.

	MGRS-A (*n* = 94)	MGRS-NA (*n* = 30)	p-value
**Treatment, n (%)**			**0.01**
Untreated	26 (27.7 %)	17 (56.7 %)	
1 Line	25 (26.6 %)	10 (33.3 %)	
2 Line	65 (69.2 %)	2 (22.2 %)	
3 Line	3 (3.2 %)	3 (10.0 %)	
**Hematologic response**			0.24
CR	8 (8.7 %)	0 (0 %)	
VGPR	16 (17.4 %)	3 (10 %)	
PR	18 (19.6 %)	7 (23.3 %)	
NR	50 (54.3 %)	20 (66.7 %)	
**Renal response**			0.42
Renal response	24 (26.1 %)	10 (33.3 %)	
Renal progression	68 (73.9 %)	20 (66.6 %)	

CR, Complete Response; aCR, CR Criterium for AL; VGPR, Very Good Partial Response; PR, Partial Response.

### Predictors for renal survival

Initial renal replacement therapy (*p* = 0.007), hypertension (*p* = 0.023), serum creatinine (*p* = 0.010), and cardiac involvement (*p* = 0.029) were found to be associated with ESRD. These statistically significant variables with p-values *<* 0.05 identified from the univariate Cox regression analysis were included in the multivariate analysis, and the authors found that hypertension (*p* = 0.005), increased serum creatinine (*p* = 0.002), and cardiac involvement (*p* = 0.022) were independently associated with worse renal survival (Table 4).

**Table 4:** Cox regression of predictors for renal survival in MGRS.

Variables	Univariate		Multivariate Analysis	
	HR (95 % CI)	p-value	HR (95 % CI)	p-value
Age	1.039 (0.995‒1.084)	0.083		
Male gender	0.622 (0.260‒1.490)	0.287		
Hypertension	0.287 (0.098‒0.844)	0.023	0.155 (0.042‒0.567)	0.005
Diabetes mellitus	0.344 (0.078‒5.490)	0.439		
Initial KRT	2.817 (1.328‒5.974)	0.007		
Serum creatinine (μmoL/L)	1.001 (1.00‒1.002)	0.010	1.002 (1.001‒1.004)	0.002
Hemoglobin	1.002 (0.998‒1.016)	0.773		
Proteinuria, g/d	1.038 (0.987‒1.093)	0.150		
Cardiac involvement	2.175 (1.084‒4.363)	0.029	2.352 (1.134‒4.879)	0.022
Nervous system involvement	0.043 (0‒10.671)	0.263		

*p* < 0.05 will be included in multivariate analysis. A p-value of less than 0.05 was considered statistically significant.

### Predictors for patient survival

Age (*p* = 0.011), cardiac involvement (*p* < 0.010), and nervous system involvement (*p* = 0.042) were found to be associated with higher overall mortality. These statistically significant variables with p-values *<* 0.05 identified from the univariate cox regression analysis were included in the multivariate analysis, and the authors found that age (*p* < 0.001) and cardiac involvement (*p* < 0.001) were independently associated with poor patient survival (Table 5).

**Table 5:** Cox regression of predictors for patient survival in MGRS.

Variables	Univariate		Multivariate Analysis	
	HR (95 % CI)	p-value	HR (95 % CI)	p-value
Age	1.075 (1.017‒1.1136)	0.011	1.068 (1.011‒1.128)	<0.001
Male gender	0.527 (0.172‒1.614)	0.262		
Hypertension	2.422 (0.793‒7.399)	0.121		
Diabetes mellitus	2.552 (0.730‒8.925)	0.142		
Serum creatinine (μmoL/L)	0.999 (0.998‒1.001)	0.344		
Hemoglobin	0.998 (0.982‒1.015)	0.851		
Proteinuria, g/d	1.044 (0.993‒1.098)	0.093		
Cardiac involvement	6.898 (2.585‒18.411)	<0.001	6.33 (2.354‒17.024)	<0.001
Nervous system involvement	0.042 (0‒42.059)	0.042		

*p* < 0.05 will be included in multivariate analysis. A p-value of less than 0.05 was considered statistically significant.

## Discussion

MGRS is a benign or precancerous hematologic disorder, but the effects on the kidneys are not benign, and patients with MGRS often develop progressive kidney disease and End-Stage Kidney Disease (ESKD). This study provides a comprehensive analysis of various aspects of MGRS, offering valuable insights into its demographic patterns, disease characteristics, laboratory findings, treatment responses, and prognostic factors. By analyzing relevant research and clinical experiences, the authors aim to provide a detailed understanding of MGRS and its prognostic factors.

The diagnosis of MGRS involves a combination of clinical evaluation, laboratory tests, and kidney biopsy.^2^ Kidney biopsy is crucial to establish the renal pathology and identify the specific type of MGRS.^6^^,^^19^ The biopsy incidence of renal amyloidosis in the studied center was 1.5 %, MGRS is associated with a wide spectrum of renal pathology, and the most common form is AL amyloidosis (75.8 % in the studied cohort), with a median age of 64.0 ys. Said SM, etc. from Mayo Clinic reported that renal amyloidosis accounted for 2.1 % of all renal biopsies with a median age of 63 years, in which AL/AH/AHL accounted for 85.9 % of MGRS.^15^ Zhihong Liu etc. reported that amyloidosis accounted for 65.5 % of MGRS in Nanjing, China, from January 2013 to December 2017.^20^ The present result was similar to the incidence of America^21^ and Western Europe,^22^^,^^23^ however, another report from Latin America indicated the most common MGRS related lesion was Proliferative Glomerulonephritis with Monoclonal Immunoglobulin Deposits (PGNMID) (33 %), amyloidosis in 26 % with limited sample size,^24^ whether there are regional characteristics requires more large-scale data center verification.

The differences in presentation between the MGRS-A group and MGRS-NA, such as a higher rate of nephrotic syndrome and lower rates of acute and chronic kidney disease in the MGRS-A group, suggest distinct pathophysiological processes or disease stages within the MGRS spectrum. In patients with MGRS-NA, renal disease was more severe compared to those with MGRS-A. A greater proportion of patients in the MGRS-NA group had elevated creatinine levels and lower estimated Glomerular Filtration Rate (eGFR). Moreover, hemodialysis was required more frequently in MGRS-NA patients than in those with MGRS-A. The lack of significant differences in free light chain ratio between the groups implies that this may not be a discriminatory factor among different MGRS subtypes. κ light chain paraprotein was higher in MGRS-NA than in MGRS-A, consistent with a report from Zi-hao Yong etc.^14^ Previous studies reported that IgG and IgA were the predominant heavy chain isotypes of M protein in patients with immunoglobulin-related amyloidosis, which was consistent with previous results.^6^^,^^20^^,^^24^, ^25^, ^26^ The findings from bone marrow biopsy, such as a lower plasma cell percentage in amyloidosis patients, suggest differences in the underlying bone marrow pathology, which could have implications for understanding the source of the abnormal immunoglobulins. Patients with amyloidosis have higher eGFR levels, higher 24-hour urine protein levels, and lower serum albumin levels, similar to previous studies.^20^ Studies have shown that patients with IgM-MGRS the most common renal disease, cryoglobulinemic glomerulonephritis, are more likely to have anemia, similar to the present result.^21^

The treatment of MGRS is aimed at both suppressing the underlying monoclonal gammopathy and managing the renal manifestations.^7^^,^^27^ The use of first-line plasma cell clone-directed chemotherapy, including bortezomib, melphan, lenalidomide, and thalidomide and/or stem cell transplantation in a significant proportion of patients shows the current treatment approach.^25^^,^^26^ However, the relatively low overall renal response rate (26.1 % in MGRS-A and 33.3 % in MGRS-NA) and hematology response rate (8.7 % in MGRS-A and 0 % in MGRS-NA) highlight the challenges in achieving optimal treatment outcomes.

Among patients who performed bone marrow biopsy, the authors found that patients with amyloidosis had lower plasma cell percentages, with 33.3 % of patients in the MGRS-NA group having a bone marrow plasma cell ratio > 10 %. There are more plasma cell malignant diseases in the MGRS-NA group, so the hematological remission rate in the MGRS-NA group was lower, while in the MGRS-A group, renal proteinuria was more prominent, so the overall renal remission rate was lower than MGRA- NA group.

The prognosis of amyloidosis-related MGRS varies depending on multiple factors including the extent of organ involvement, response to treatment, and the underlying type of amyloidosis. Early diagnosis and initiation of appropriate treatment are key to improving outcomes. However, there is still a lack of large-scale prospective studies to establish definitive treatment guidelines and accurately predict prognosis. The authors found that both hematologic and renal responses are associated with longer survival emphasizing the importance of effective treatment in improving prognosis, consistent with another report from Alessandro Gozzetti etc.^14^^,^^28^ The lower renal survival in the MGRS-A group compared to MGRS-NA indicates the more renal-aggressive nature of amyloidosis-associated MGRS and the need for more aggressive treatment strategies in this subgroup.

For renal survival, hypertension, increased serum creatinine^28^ and cardiac involvement were independent risk factors for ESKD in MGRS. This highlights the need for close monitoring of these parameters and targeted interventions to improve outcomes. In terms of patient survival, age and cardiac involvement are significant predictors. The identification of these factors emphasizes the importance of considering the patient's overall health status and organ involvement in the management and prognosis of MGRS. The above indirect evidence and the literature review support the importance of cardiac-related mortality in MGRS-A.

The study had limitations. First, as a retrospective study of a rare disease, prospective sample size estimation was impractical. However, post-hoc power analysis confirmed adequate statistical power (92 %) to detect clinically relevant effect sizes; residual risks of spectrum bias and unmeasured confounders persist in observational designs. Second, the duration of follow-up was relatively short; the long-term follow-up of these patients is essential to monitor the progression of the disease and the response to therapy. Furthermore, single-center recruitment introduces institutional selection biases in treatment protocols and diagnostic workflows, though standardized histopathological review mitigated interpretation variability.

This large-scale cohort study establishes amyloidosis as the predominant pathology (75.8 %) driving Monoclonal Gammopathy of Renal Significance (MGRS) in Western China, revealing critical risk determinants ‒ particularly cardiac involvement and serum creatinine elevation ‒ that independently predict ESKD. These findings underscore the urgent need for early hematologic intervention in MGRS-A, given the profound survival benefit linked to treatment response.

## Informed consent statement

This investigation followed the medical ethics guidelines stated in the Declaration of Helsinki and was authorized by the Ethics Committee of Sichuan Provincial People’s Hospital. All the participants were informed about the research before acquiring their consent.

## Funding

No funding.

## Data availability

The data that support the findings of this study are available from the corresponding author upon reasonable request.

## CRediT authorship contribution statement

**Junru Wang:** Conceptualization, Methodology, Formal analysis, Writing – original draft. **Kun Peng:** Investigation, Data curation, Resources, Visualization. **Guisen Li:** Validation, Writing – review & editing, Project administration. **Shasha Chen:** Supervision, Funding acquisition, Writing – review & editing.

## Declaration of competing interest

The authors declare that they have no known competing financial interests or personal relationships that could have appeared to influence the work reported in this paper.
